# Patient, caregiver, and health care provider perspectives on barriers and facilitators to heart failure care in Kerala, India: A qualitative study

**DOI:** 10.12688/wellcomeopenres.16365.2

**Published:** 2021-04-19

**Authors:** Prinu Jose, Ranjana Ravindranath, Linju M. Joseph, Elizabeth C. Rhodes, Sanjay Ganapathi, Sivadasanpillai Harikrishnan, Panniyammakal Jeemon

**Affiliations:** 1 Public Health Foundation of India, New Delhi, India; 2 Sree Chitra Tirunal Institute of Medical Sciences and Technology, Trivandrum, India; 3 Center for Chronic Disease Control, New Delhi, India; 4 University of Birmingham, Birmingham, UK; 5 Yale Center for Implementation Science, Yale School of Medicine, Connecticut, USA; 6 Department of Social and Behavioral Sciences, Yale School of Public Health, Connecticut, USA; 7 Center for Methods in Implementation and Prevention Science, Yale School of Public Health, Connecticut, USA

**Keywords:** Heart failure, Kerala, lifestyle modification, self-management, quality of care, qualitative

## Abstract

**Background: **Deficits in quality of care for patients with heart failure (HF) contribute to high mortality in this population. This qualitative study aimed to understand the barriers and facilitators to high-quality HF care in Kerala, India.

**Methods: **Semi-structured, in-depth interviews were conducted with a purposive sample of health care providers (n=13), patients and caregivers (n=14). Additionally, focus group discussions (n=3) were conducted with patients and their caregivers. All interviews and focus group discussions were transcribed verbatim. Textual data were analysed using thematic analysis.

**Results: **Patients’ motivation to change their lifestyle behaviours after HF diagnosis and active follow-up calls from health care providers to check on patients’ health status were important enablers of high-quality care. Health care providers’ advice on substance use often motivated patients to stop smoking and consuming alcohol. Although patients expected support from their family members, the level of caregiver support for patients varied, with some patients receiving strong support from caregivers and others receiving minimal support. Emotional stress and lack of structured care plans for patients hindered patients’ self-management of their condition. Further, high patient loads often limited the time health care providers had to provide advice on self-management options. Nevertheless, the availability of experienced nursing staff to support patients improved care within health care facilities. Finally, initiation of guideline-directed medical therapy was perceived as complex by health care providers due to multiple coexisting chronic conditions in HF patients.

**Conclusions: **Structured plans for self-management of HF and more time for patients and health care providers to interact during clinical visits may enable better clinical handover with patients and family members, and thereby improve adherence to self-care options. Quality improvement interventions should also address the stress and emotional concerns of HF patients.

## Background

Globally, more than 37.7 million people suffer from heart failure ^1^. As the prevalence of cardiovascular diseases (CVDs) continues to rise rapidly in low- and middle-income countries (LMICs) ^2^, the burden attributable to HF is likely to increase in these settings. India accounts for a major share of the global burden of HF with an estimated 4 million cases ^3^, and unlike many individuals with HF from high-income countries, Indian adults are often affected in their most productive years of life ^4^. Within India, the state of Kerala is in the advanced stage of demographic and epidemiological transitions with relatively high life expectancy and disease burden from chronic non-communicable disease conditions ^4^. In Kerala, CVD is the leading cause of death ^4^, and HF is a major contributor to CVD mortality. Further, data from clinical registries from Kerala indicate that almost one in two patients die within a three-year period from the date of their original admission to a health care facility for HF ^5^.

Heart Failure is recognised as a major public health problem due to the high mortality associated with the condition, devastating effects on patient’s lives including productivity loss ^6^, quality of life, high health care utilisation due to frequent hospitalisation, and associated catastrophic health spending in LMICs. The high mortality burden can be mitigated by addressing hospital readmissions and sub-optimal adherence to Guideline Directed Medical Therapy [GDMT] among HF patients ^5^. Poor adherence to medication and other self-management options, sub-optimal clinical hand-over (defined as the transfer of professional responsibility and accountability for some or all aspects of care for a patient, or group of patients, to another person or professional group on a temporary or permanent basis ^7^), multi-morbidity and associated emotional stress are important reasons for poor quality of life in patients with HF ^8– 13^. Being a progressive disease, HF can impair a patient’s ability to practice self-management ^14^ and perform daily activities ^15^. Moreover, limited understanding of the condition and treatment often affects the capacity for self-management in HF patients ^16^. Given the chronic nature of the condition, optimal self-management of HF requires involvement of caregivers at home ^15^. However, caregivers’ inadequate knowledge and skills to support self-care for patients with HF ^17, 18^ is a major concern as they are not fully involved in treatment and management decision making process.

Findings from a prior qualitative study in Kerala with health care providers highlighted the need for a comprehensive quality improvement initiative to improve HF care ^19^. The study reported that while health care providers have focused on acute coronary syndromes, inadequate attention has been given to HF. According to health care providers’ perspectives, specialised cardiac services were not available, discharge education was inadequate, and provisions of guideline-directed medical therapies for patients with HF was sub-optimal ^19^. The perspectives of patients and their caregivers regarding the difficulties patients face in managing their condition are yet to be explored.

To gain a more comprehensive understanding of the barriers and facilitators to HF care in Kerala, it is essential to explore the perspectives of health care providers, the users of HF care and their caregivers. In this study, HF care refers to the care provided in health care facilities as well as the self- management of HF patients. Qualitative research methods are useful to gain insights on the experiences, needs, and challenges to care in patients with CVD ^20, 21^. In this paper, we aim to describe perceptions on facilitators and barriers to HF care by utilizing qualitative research methods in Kerala, India. A comprehensive understanding of the perspectives of patients, caregivers, and Health Care Providers (HCPs) may help to identify the existing challenges of HF care and health care utilization. Further, as HF patients and caregivers are important stakeholders of HF care, considering their views and opinions in the design of quality improvement interventions can help ensure interventions meet their needs.

## Methods

### Ethical considerations

The Institutional Ethics Committee of Sree Chitra Tirunal Institute for Medical Sciences and Technology, Trivandrum provided ethical approval for this study (Approval letter: SCT/IEC/1313.10 dated 15/12/2018). We explained the study purpose to all participants and obtained their written informed consent to participate. All interviews and focus group discussions were conducted in a private location, and all participants were informed of their right to withdraw from the study at any time. All transcripts were de-identified to ensure anonymity of participants.

### Setting and study sites

The study was conducted in the HF clinic of Sree Chitra Tirunal Institute for Medical Sciences and Technology (SCTIMST), which is a tertiary care referral center (public clinic) in Trivandrum, Kerala. We also included HCPs from other leading private hospitals in Kerala in our sample. 

### Study participants, recruitment, and sampling

Study participants included different stakeholders of HF care, including patients, caregivers, and HCPs. Semi-structured, in-depth interviews were conducted with all stakeholders, and focus group discussions (FGDs) were conducted with patients and caregivers. Purposive sampling ^22^ was used to recruit patients and caregivers across age and gender groups to obtain a diverse study group. A gatekeeper strategy ^23^ was utilized by recruiting patients and caregivers with the help of a research nurse employed in the HF clinic. Patients who were waiting for the follow-up clinical consultation in the HF clinic were invited to participate in the study. All patients who were aged 18 years or older and attended the HF clinic for clinical follow-up were eligible to participate. Patients who were deemed not well enough to participate (defined as HF patients who presented with acute symptoms like shortness of breath) were excluded, considering the need for immediate medical attention. Patients who were next in line for a doctor consultation also were excluded to avoid interruptions to consultations in the HF clinic. Primary caregivers who accompanied patients for hospital consultations were also invited to participate in the study. When a patient was accompanied by more than one caregivers, the primary caregiver was chosen as the person who is most involved in care, as identified by the patient.

To further explore the challenges and facilitating factors for HF care, we recruited doctors and nurses (referred as “HCPs”) employing two recruitment strategies. Firstly, purposive sampling ^22^ was employed to identify information-rich individuals with a diversity of characteristics (e.g., type of health care provider). We also included HCPs who were experienced cardiologists working in private hospitals of central Kerala. Secondly, to identify HCPs with specific experience in HF management, snowball recruitment ^24^ was employed by asking HCPs at the end of interviews to refer other HCPs. They were subsequently contacted and invited to participate in the study.

### Data collection, quality control, and management

First, FGDs were conducted with the HF patients and caregivers. Then, to gain a deeper understanding of these issues, we conducted face-to-face in-depth interviews (IDI) with patients, caregivers, and HCPs. The interview guides for patients and caregivers were translated to the regional language Malayalam. All data collection instruments were semi-structured guides tailored to the type of stakeholder and designed to elicit information on health behaviours, difficulties in care accessibility, caregiver’s role, and acceptability of a nurse led HF program. The HCPs were interviewed to understand the impact of the disease in patients and families with questions on perceived quality of HF management. All the participants were asked the same key questions in the interview guide with minor refinements to understand relevant issues specific to each participant. Full interview and FGD guides are available as *extended data* ^25^

Data were collected from June 2019 to February 2020 by the first author (PJ) who has a master’s degree in public health, training and experience in qualitative research, and experience conducting research on cardiovascular risk reduction. Additionally, three research nurses assisted PJ in data collection process by conducting interviews and note taking for FGDs. One assistant worked in the HF clinic and two other assistants worked as research nurses in cardiovascular risk reduction projects. Out of four interviewers, three were female researchers.

All the interviewers and moderators were native Malayalam speakers. The interviewer introduced themselves, explained the purpose of the project, and acquired written informed consent to participate in the study from each participant. Interviews and FGDs were conducted in private, quiet locations inside the HF clinic or waiting areas adjacent to the clinic. All interviews were audio-recorded and lasted approximately 30 minutes to one hour, except for two, that were shorter in duration due to logistical issues. Interviews with patients and caregivers were conducted in Malayalam. Interviews with HCPs were conducted in their preferred language, either English or Malayalam. Participant names, transcripts and audio files were stored on a password-protected computer. The audio files and transcripts will be archived for five years from the completion of the study. The Consolidated Criteria for Reporting Qualitative Research standards (COREQ) - a 32 item checklist - was used to guide the reporting of this study ^26^ and is available as *reporting guidelines* ^27^.

### Data analysis

All audio recorded interviews and FGDs were transcribed verbatim. The interviews conducted in Malayalam were simultaneously translated from Malayalam to English and transcribed. The deidentified transcripts are available as *underlying data* ^28^ *.* PJ reviewed the full transcripts while listening to the audio recordings to ensure quality of transcription. For data compilation and analysis, data were imported into MAXQDA Standard 2018 (Release 18.2.3). Data were analysed using thematic analysis ^29^ by hybrid approach which incorporates the data driven approach from Boyatzis, 1998 ^30^, and the deductive a priori template of codes approach by Crabtree and Miller, 1999 ^31^. The coding manual of this study comprised of inductive codes which emerged from data and deductive codes of topics from the interview guide ^29^. Members of the team analysing the data (PJ and RR) read all of the transcripts closely to familiarize themselves with the data *(Stage 1).* The codes were organized into a coding manual which included the code name, code definition, and examples of textual data. The examples were provided to help ensure that the codes were applied properly. The coders (PJ and RR), met frequently throughout the coding process to discuss and any differences was resolved by a third qualitative researcher (LJ) who reread the underlying data *(Stage 2)*. At this stage, the initial coding manual was developed *(Stage 3).* This coding manual was then applied to the remaining transcripts and relevant segments were assigned with suitable codes using the code system in the software. Further, we added data-driven inductive codes, which were missed in the initial coding manual *(Stage 4).* Additionally, memos were made to note the thoughts regarding each segment throughout data analysis. All the codes were then organized according to broader barrier and facilitator themes. After discussing with the team members, the final themes were developed and underpinned meanings were defined *(Stage 5 and 6)* ^29^ *.* Finally, a social-ecological model ^32^ was utilized to organize themes on barriers and facilitators in patient, caregiver and HCP levels. The researchers practiced reflexivity throughout the study and remained sensitive to the ways in which their experiences and prior assumptions shaped the findings.

## Results

### Participant characteristics

We conducted two FGDs with patients (six patients in each FGD) and one FGD with caregivers (five caregivers). Additionally, 10 and four in-depth interviews were conducted with patients, and caregivers, respectively. We also conducted 13 in-depth interviews with health care providers. Most of the patients (19 of 22) were males with age ranging from 36–65 years. The mean age of the HF patients in the study was 60.86 ± 11.30 years. The routine follow-up period of each patient ranged from once in two-months to one-year. Most caregivers who participated in the study were females. Among health care providers, most (11 of 13) were employed by Government-funded public sector hospitals. Most doctors (5 of 7) were consultant cardiologists, and the rest were senior residents in the Department of Cardiology. All nurses had experience in managing and treating HF patients in either the intensive care units or HF clinics. Demographic characteristics of the participants are provided in Table 1.

**Table 1.** Demographic characteristics of in-depth interview participants.

Participants	N (%)
***Type of health care provider***	
Total number	13
Cardiologists	5 (38.4%)
Senior Residents	2 (15.3%)
Nurses	6 (46.3%)
***Patient characteristics***	
Total number	22
Male	19 (86.3%)
Age (range)	34 –77 years
***Caregiver characteristics***	
Total number	9
Male	1 (11.1%)

### Study themes

We identified a total of nine themes relating to the barriers and facilitators of HF care, which were grouped into patient, caregiver and HCP levels. For patients, motivation to improve lifestyle behaviours after HF diagnosis and follow-up calls from hospital staff were key enablers. However, emotional stress and lack of clear care plans for self-management were important challenges to their HF care. Caregiver support was perceived as a facilitating factor and barrier to HF care. Even though patients expected support from their family members, the level of caregiver support for patients varied, with some patients having strong support from caregivers and others receiving minimal support. Health care providers viewed the initiation of guideline-directed medical therapy as challenging due to suboptimal health status of patient. Further, high patient loads for HCPs often limited the time for providing one-to one self-management advice to patients. However, involvement of HCP in behavioural modification and availability of experienced nursing staff helped improving HF care. These themes are provided in Table 2.

**Table 2.** Key themes and illustrative quotes in patient, caregiver and health care provider levels.

Level	Themes	Illustrative quotes
**Patient** **level**	Motivation to improve lifestyle behaviour post-diagnosis	“If meat is bought at home, I will not eat it because my cholesterol level might rise.” -FGD Patients, Male
	Follow-up calls from hospital staff helping patients	“A doctor called me and introduced himself as the assistant of [Name of treating doctor]. I was at home. He enquired me whether I came for the follow-up and asked about my health condition.” -IDI Patient,72 years, Male
	Emotional stress	“Patients are mental distressed due to this illness. They will be depressed. I think young people might feel depressed than others” -IDI Patient, 68 years, Male
	Lack of clear detailed care plans for self-management	“The doctor asks whether I exercise regularly and I answered him accordingly. However, they have not taught me any specific exercise yet.” -IDI Patient,63 years, Female
**Caregiver** **level**	Caregiver support	“Husband and daughter always remind me to take medicines at right time, even if I forget they are conscious about that and always reminds me” -IDI Patient, 68 years, Female “I think it is better not to tell about it. No one is supportive at home” -IDI Patient, 72 years, Male
**Health care** **provider** **level**	Advice on substance use	“In the previous visit, they advised me to stop drinking alcohol. I used to drink a small quantity of alcohol before.” -FGD Patients, Male
	Availability of experienced nursing staff	“Yes, there is huge gap which can be partly filled by the doctor but mainly by the supporting staff. I think there lies the importance of a heart failure nurse, who will work with you so that he/she will spend time for proper rehabilitation of the patient. -IDI Doctor, Public clinic
	Perceived value and usage of Guideline Directed Medical Therapy (GDMT) in clinical practice	“We are not that good that is what our data shows. All the data that we have collected showed that only 1/3rd of the patients get the guideline directed therapy.” -IDI Doctor, Private clinic
	High patient caseload	“The time allotted or spend by doctors with patients will not be good enough at times. In the west, they can’t see more than 10 patients per day. So, time spend with patients will be more.” -IDI Doctor, Public clinic

FGD – Focus group discussion, IDI - In-depth interviews

### Patient level

***Motivation to improve lifestyle behaviours post-diagnosis*.** Many patients were motivated to improve their lifestyle behaviours after being diagnosed with HF because they recognized that healthy lifestyle behaviours were important for maintaining their health and preventing the exacerbation of HF. Many patients reported that they started daily walking exercise. In particular, patients acknowledged the need for a healthy diet to avoid health complications in the future, such as an increase in blood sugar, blood pressure or cholesterol levels. Further, many patients shared that they were successful in modifying their diets. Some patients reported that they stopped or reduced their intake of foods they believed could lead to higher blood cholesterol, such as sugar and non-vegetarian food items like meat and chicken. Additionally, most patients emphasized that they switched from a predominantly rice-based diet to a wheat-based one. Further, some participants also reduced their portion sizes.

*“I used to eat a lot meat, fish and all. Now, I stopped all that. I eat fish; however, the quantity is less. Also, I have reduced the amount of rice in the afternoon. At night it will be wheat.”*

                                                                        (IDI, Patients, 39 years)

However, in one case, a patient’s caregiver shared that their household was following a naturopathy diet because of a HF diagnosis. Naturopathy is an alternative system of medicine that directs patients to eat a predominantly vegetarian diet with less salt, oil, and masala (spices) for treatment and disease prevention.

***Follow-up calls from hospital staff helping patients*.** Some patients noted that health care providers called them after their out-patient visits to check on their current health status. Notably, when patients were asked about their concerns regarding the small number of follow-up visits, they stated that as staff enquires and provides feedback by telephone regularly, they felt reassured. Some patients believed that it is better to avoid recurrent hospital visits when they are asymptomatic.

*“When there are no issues, there is no need to come frequently to the hospital. It will be difficult for the hospital as there are many patients who are waiting for their consultation day. Moreover, the staff contacts us through phone. As they take our feedback here, there is no problem. Usually, counsellors and the doctor will call me. They ask about the condition and enquire whether there is any problem in doing my regular job. It is really good.”*

                                                                        (IDI Patient, 52 years)

However, in one case, a patient thought that HCPs called to determine whether he was alive. Rather than reassurance, he felt that the HCPs merely contacted to understand his health condition and follow-up status.

The HCPs confirmed that telephone follow-up was encouraged, as they were concerned about the inability to consult with patients once in a month or two months in clinics due to high patient caseloads. Therefore, some patients were contacted by the hospital staff to understand the patient’s health condition over phone. The HCPs believed that telephone calls improved not only the rapport between patients and hospital but also the regularity of patient follow-up.

*“Previously there were three to four nurses working here. So, they had a lot of time dedicated to the program. They used to call these patients, periodically ask them about taking medications and advise them on restricting salt, restricting fluids, and regularly testing the blood. This made a lot of difference.”*

                                                                        (IDI Doctor, Public clinic)

***Emotional stress*.** Most patients and their caregivers felt highly stressed. A major source of stress was financial problems, which patients and caregivers directly linked to HF treatment costs and the chronic nature of the condition. Patients expressed concern about struggling to manage given their low incomes and the high cost of treatment. They also were anxious about not being able to afford some of their prescribed medications because they were costly and not available through ‘Jan Aushadhi’ outlets, which are government owned medical stores providing medicines at affordable prices.

*“I am tensed about medication usage. We have to consume these medicines continuously, right? Also, some patients might be unable to afford the cost of these medicines. Certain prescribed medications are not commonly available.”*

                                                                        (IDI Patient, 74 years, Male)

The HCPs underscored that HF patients were often stressed because of the lack of affordable medication (for example, novel drugs like angiotensin receptor - neprilysin inhibitor) and costs of treatment. They also considered patients’ inability to work due to their condition to be a source of stress for patients. Additionally, some patients were anxious about the early onset of HF given that it coincided with the most productive period of their lives. The HCPs perceived that HF patients were worried about the chronicity and incurable nature of the disease. Moreover, HCPs felt that female caregivers were mostly stressed due to the disrupted financial situation as they were unable to earn as much to fulfil the needs of the whole family.

*“If they are male, they might be the earning member of the family. If he is unable to go for job, his wife has to go. She might not earn that much. She will be depressed as she has to take care of the patient and their children”*

                                                                        (IDI Nurse, Public clinic)

Patients felt that their caregivers were more stressed than themselves because of a sudden alteration in the traditional gender roles of the primary earning family member and additional familial responsibilities. Women who cared for their husbands after HF described particularly high levels of stress, as they needed to become the primary financial providers in their households in addition to continuing their traditional role of household cooking, cleaning, caring for children, etc. Some female caregivers reported that they were struggling to fulfil the responsibilities as they must balance both caregiving and provider roles within the family.

*“My husband is a driver. Now, he is not having a job and I do whatever work I get. The doctor suggested him not to drive. So, I cannot send him for any job. Understanding my financial condition, some of my friends send money once in a while. I am managing my son’s education with that money. Even though he is studying in a government school, we still have to buy uniforms and books.”*

                                                                        (IDI Caregiver, Female)

*“Caregivers are playing the most important role. They will be in a dilemma as the patient might be unable to play the familial and earning member role. Caregivers have to do all of these roles now. So, they should be given courage. At times, they have to take the complete responsibility of the family.*

                                                                        (IDI Patient, 63 years, Male)

***Lack of clear and detailed care plans for self-management*.** Some patients reported that while it was common for HCPs to advise them to exercise, clarity regarding how to exercise was often lacking. For instance, patients recounted that during clinical consultations HCPs did not provide instructions on specific exercises to do or demonstrate possible types of exercises that can be done after a HF diagnosis. Similarly, patients reported that although HCPs often encouraged them to improve their diet by reducing salt, sugar, and oil, specific instructions on ways to eat healthier were not discussed during the clinical consultation. Patients believed that they were not given more detailed advice because HCPs were busy and had limited time to communicate with each patient. The HCPs acknowledged that they did not have an effective and comprehensive rehabilitation program for HF patients, especially after their discharge from clinics.

*“Because unfortunately we don’t have a plan for transition from hospital to home which is an important step.”*

                                                                        (IDI Doctor, Public clinic)

Furthermore, HCPs highlighted the limited availability of cardiac rehabilitation services in the state of Kerala, noting that services like HF rehabilitation and patient-oriented exercise programs was considered non-existent and has been given very little attention. Although there were programs to support people who have suffered from heart attacks, specialised rehabilitation services for HF were unavailable.

*“As far as rehabilitation facilities are concerned, to be frank we don’t grade it according to the patient. That is a significant gap in our management because we never tell any patient how much or what is the intensity of the exercise or walking which they can do. We will just tell them to go for walking if at all he is a diabetic and adjust his medications and then diet control”.*

                                                                        (IDI Doctor 4, Public clinic)

### Caregiver level

***Caregiver support*.** Caregiver support was both a facilitator and barrier to HF care. Among patients, support from caregivers was viewed as necessary for HF management. Some patients described that their family members helped them with cooking and daily household responsibilities such as washing clothes and drawing water from well. Patients also shared that their family members did not allow them to do activities that are physically exhausting, such as ploughing land and cutting wood. They recognized that this support was important because they were unable to engage in strenuous activities after HF.

*“Also, if I want, I can cut wood, dig and all. But family will not allow me to do it. I am not lazy to cut wood. But they won’t allow me. They will wash even my clothes. My wife does all that for me”.*

                                                                        (IDI Patient, 72 years)

Additionally, patients noted that caregivers managed their hospital records and accompanied them to their clinic appointments. Several patients shared their support from a caregiver for regular medication intake. Patients noted that caregivers reminded them to consume medicines or handed them the prescribed medicines and ensured their intake. Health care providers recognized and appreciated the role caregivers played in assisting HF patients. Further, they emphasized the importance of educating caregivers on HF and best practices for helping patients manage their condition. Patients also described ways in which caregivers provided economic support (for example, many patients shared that their family members paid for their treatment and medications) and emotional support (for example, patients described that caregivers reassured and frequently enquired about their health status). In one case, a female HF patient stated that more than medications, the mental support from family members was helping her in managing the disease.

However, some patients stated that their caregivers would be unavailable at home due to educational needs or job responsibilities. Health care providers shared similar views regarding the availability of caregivers.

*“But most of the people go out for work, they will not be available throughout the day. Especially during office hours, they may not be available. They may be going to school or college or they will be going for work.”*

                                                                        (IDI Doctor, Public clinic)

Some patients felt that they did not receive adequate support from caregivers with regards to their disease management. In one case, a patient perceived that his family members were not supporting him adequately, which caused him extreme distress.

*“I am unable to sleep at night. People whom I worked for all these years do not care for me now. It is very painful*. *There is no one to help me.”*

                                                                        (IDI Patient, 64 years)

### Health care provider level

***Advice on substance use*.** Receiving advice on substance use from HCPs strongly influenced patients to modify their lifestyle. Patients reported that instructions from HCPs about the importance of smoking cessation and abstinence from alcohol motivated them for cessation, and they described their experiences of quitting smoking and alcohol use after receiving advice from HCPs.

*“At the age of 40, when a nurse or doctor tell us to stop smoking or else you will die, you will definitely stop. Even now, if I accompany anyone for a tea and Wills (cigarette), I used to keep the cigarette in my mouth unknowingly. People ask me whether I smoke. If I smoke a cigarette again, then all that I gained after quitting smoking will be ruined”*

(IDI Patient, 68 years) ( *Patient was chronic smoker who started smoking at an early age*)

The HCPs reported that they educated patients and their families about the importance of quitting smoking and reducing alcohol consumption. Nurses also specifically noted that they provided detailed advice on smoking and alcohol cessation during each patient’s hospital visit or as a part of discharge education.

*“We educate about the weight maintenance, avoidance of smoking and about the do’s and dont’s on diet.”*

                                                                        (IDI Nurse, Public clinic)

***Availability of experienced nursing staff*.** Many physician consultants recognized that having experienced nursing staff available to support HF management was key for high quality care. They acknowledged the ability of experienced nurses to promptly understand patients’ HF symptoms and communicate with patients effectively. Further, they believed that nurses were able to build and maintain rapport with patients.

*“[We have] experienced consultants to experienced nursing staff. It is more important. If something suddenly goes wrong with a patient in ICU, the nursing staff will identify. That is very good in this hospital. When you compare the paramedical staff, they are very alert. They will call us and inform if a patient’s condition goes bad”.*

                                                                        (IDI Doctor, Public clinic)

Patients often sought help with HF management from nurses and were generally receptive to following advice provided by nurses, as they felt nurses were more approachable than doctors. One doctor shared his experience about the role of “nurse practitioners” who assist doctors in patient decision making. In foreign countries, nurse practitioners and doctors work as a team when developing the patient care plan. The nurse practitioners often ensure that there is no delay in making treatment decisions for patients.

*“The nurse practitioner always assists doctors, especially for in-patient care. The doctor and nurse discuss among themselves and come with a patient management plan. If the physician is not available, the nurse practitioners can make decisions for the patient’s management.”*

                                                                        (IDI Doctor, Public clinic)

***Perceived value and usage of Guideline Directed Medical Therapy in clinical practice*.** The physician consultants recognised and described the Guideline Directed Medical Therapy (GDMT) as being pertinent to HF management as it reduces the number of re-hospitalization events. Further, it can improve the overall quality of HF patient care. However, many HCPs were concerned about the current usage of GDMT for HF patients. Specifically, they emphasized that only a few patients received GDMT before discharge due to the high cost of medications, which were unaffordable to many patients and lack of awareness among HCPs about the advantages of this therapy. Further, patients often had multiple co-existing chronic conditions, which often prevented the HCPs from initiating and up titrating all the medicines under GDMT. Conversely, one HCP believed that doctors’ lack of time for each patient consultation due to busy schedules was a key reason for non-prescription of GDMT.

*“Physician may not get time to up-titrate treatment. In the busy practice they may not up-titrate treatment. Very often physician just start on giving the initial dose and refer else were. And the patient may not follow. Or if the patient follows the physician may forget to increase the dose”*

                                                                        (IDI Doctor, Private clinic)

*“Because at the time of discharge, patient may not be in an optimal state to decide on the optimal dose of medicines. Sometimes it may not be possible to start all the medicines”.*

                                                                        (IDI Doctor, Public clinic)

***High patient caseloads*.** The HCPs raised concerns over their high patient caseload, describing that they often had to treat more patients than allotted for in their schedules. The increased workload reduced the time allotted for an individual patient for consultation. They recognized that some patients were dissatisfied with the short length of the consultation and, in some cases, having to have consultations with residents instead of consultant cardiologists.

*“When I am in the OPD, all patients want to see me, but it is not possible. So, half of the patients will be seen by the residents. These patients are unhappy that they are not able to consult me”.*

                                                                        (IDI Doctor, Public clinic)

To reduce patient caseload, doctors recommended scheduling appointments with fewer patients and increasing the number of consultant cardiologists within clinics.

*“I think there should be dedicated heart failure specialist. If I have two more consultants with me, it will increase the number of patients seen and managed properly. Further, the patients will get more satisfaction*”.

                                                                        (IDI Doctor, Public clinic)

**Figure 1.**
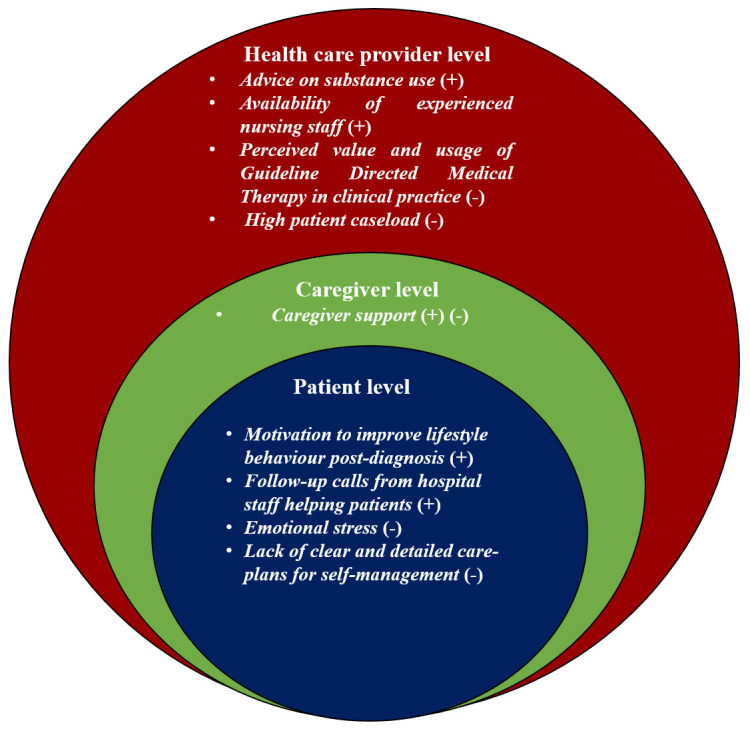
Summary of themes, presented within the social-ecological framework as described by participants. (+) indicates a facilitator for heart failure care and (-) indicates a barrier to heart failure care.

A summary of the identified themes is displayed in Figure 1.

## Discussion

We identified multiple barriers and facilitators of HF care from the perspectives of patients, caregivers, and HCPs in Kerala. Heart failure patients in general appreciated the role of caregivers and it was perceived as important for their adherence to heart failure treatment in our study. However, high patient caseload during physician consultations, non-adherence to GDMT and lack of clear self-management care plans are recognized as important barriers to optimal HF care. Another important perceived barrier to HF care is the lack of specific rehabilitation plans for self-management. Patients and their caregivers’ accounts also illustrate the altered gender roles within their family, productivity loss and the caregivers stress as barriers to HF care. The study participants agree in general that the availability of experienced nurses could help in improving the quality of care. Furthermore, direct advice from HCPs motivated patients to adhere to self-care activities and stop behaviors such as smoking and alcohol use.

In our study, patients reported that they modified their lifestyle only after the HF diagnosis. The change can be possibly due to the advice received from HCP’s and fear of potential complications of HF. Further, patients might have acknowledged the importance of adherence to a healthy lifestyle foreseeing its positive health outcomes. This is consistent with previous studies, which noted that good motivation and fear of disease complication were associated with adherence to lifestyle change for people with and at risk of cardiovascular diseases ^33, 34^. Follow-up phone calls from HCPs especially nurses helped in building a good rapport with the HF patient. The follow up calls reinforced the need for healthy behaviours like regular follow-up visits and adherence to medication and other self-care activities. Consistent findings reported in other studies have highlighted that mobile health (mHealth) services enabled nurses to improve cardiac outcomes in HF patients ^19, 35^.

Even though patients were motivated and willing to improve their lifestyle behaviors, there were no specific self-management plans for HF patients. Patients perceived the lack of self-management care plans as an important barrier to optimal care. The American Heart Association recommends patients with HF to have a healthy diet, regular physical activity, stress management, alcohol, and smoking cessation ^36^. Studies have shown the role of self-management as an effective approach to improve health outcomes in LMICs ^37^. However, adherence to self-care recommendations were low in HF patients from low and middle income country settings (LMIC) ^38^. Further, the current focus of the health system in improving acute management often ignores chronic long-term management of HF ^19^. Our study also highlighted the lack of cardiac rehabilitation programmes customized for HF patients. There were no explicit plans for the transition of patients from hospital to home. Lack of access to cardiac rehabilitation services due to poor availability are reported in other studies conducted in Kerala ^39^. Further, previous studies have also emphasised the importance of a systematic discharge plan as part of a formal clinical handover of health related information for patients and caregivers to improve health outcomes ^19^.

The physician consultants in our study highlighted their inability to initiate or up-titrate GDMT as indicated. Patient’s sub-optimal health due to co-existing multiple chronic conditions is highlighted as one of the important reasons for non-prescription of GDMT. Lack of awareness and busy practice with high patient load are also given as other potential reasons. The high patient load for consultants often results in poor quality of care. Shortage of specialist consultants in general for both urban and rural services in India are described in other studies ^40^. Alternative, options to improve the availability of time for interaction between patients with HCPs other than physician consultants may improve adherence to GDMT. The Trivandrum Heart Failure Registry ^41^ of 1205 HF cases in Kerala reported that GDMT was given only to 19% and 25% of patients with left ventricular systolic dysfunction during hospitalization and at the time of discharge, respectively. Quality improvement initiatives like task sharing with non-physician health workers may improve prescription and adherence to GDMT and help the patients to reach the target dose for each drugs based on evidence and clinical recommendations. For example, Strömberg *et al.* ^42^ reported that task sharing strategies such as nurse-led heart failure clinics improved the survival and reduced the number of readmissions for patients with HF.

Availability of experienced nursing staff was recognised as an important enabling factor for utilisation of optimal HF care. This is consistent with a previous study conducted in Kerala, which suggests that nurses can be “ *key leverage points*” in improving the quality of HF management ^19^. Patients in our study received advice from nursing staff regularly, which helped them in cessation of certain harmful behaviours like alcohol use and smoking.

Our study highlights that emotional distress, anxiety like symptoms and depression are major roadblocks to seek optimal care in HF patients and their care givers. Depression is one of the most prevalent co-existing chronic conditions with HF. The reported prevalence of depression in HF patients is 21.5% ^43^. The emotional distress of HF and its causes reported in our study was similar to those reported in the literature, which suggest that depressive symptoms in HF patients is not only due to the disease condition but also related to other contributing factors like the inability to work, financial constraints and poverty ^44^. Moreover, depression affects the quality of life and worsens clinical outcomes in HF patients ^45^. Moderate to severe depression has been shown to be associated with a 2-fold increase in the risk of hospitalisation and 4-fold increase in all-cause mortality in a community population living with HF ^46^. Our study noted that caregivers were distressed due to role change, financial constraints, and familial issues. Assessment and management of depression is also important in the caregivers of HF patients. Poor functional status and overall distress were reported in caregivers of patients with HF in other studies ^47^. Further, a systematic review suggests that the caregiver’s mental and physical health influence health seeking behaviours and self-care practices in HF patients ^48^. Therefore, the package of intervention in HF should include components of screening and management for depression or symptoms related to depression and anxiety.

Our study findings highlight the absence of a structured care-plan for imparting self-management skills for both patients and caregivers. The clinical handover of information is therefore incomplete from HCPs to HF patients and their caregivers. Inadequate clinical handover may lead to poor adherence to self-care activities, medications errors and disrupts continuity in care. Development of self-management skills is of utmost importance owing to the chronicity of HF and less dedicated doctor-patient time. Further, proper clinical handover empowers the HF patient and caregiver to identify the warning signs of worsening of the condition in advance and initiate timely management. Such strategies are often helpful in reducing the re-hospitalisation events and mortality. The Chronic Care Model (CCM) described by Wagner ^49^ intends to optimize the chronic disease management process. The dimensions of CCM include the community, the health system, self-management support, delivery system design, decision support, and clinical information system. Effective linkages with community resources such as regular interaction with community health workers could help the patients and families dealing with the challenges of managing chronic illness. Additionally, continuous engagement of patients with HCPs either through telephonic interactions or mHealth could produce better health outcomes in HF patients.

### Strengths and limitations

A major strength of this study is that it provides a multi-stakeholder perspective by involving HCPs, patients and caregivers. Accordingly, the findings help ensure their opinions and perspectives are prioritized in the development of future interventions to improve the quality of HF care in Kerala. In addition, we used the Consolidated Criteria for Reporting Qualitative Research standard to promote explicit and comprehensive reporting of the study ^27^. Our study also had limitations. We excluded extremely sick patients with HF. To mitigate this, we identified barriers and facilitators to care specific to sick patients through interviews with HCPs. Although we used purposive sampling to ensure inclusion of HF patients of varying ages in our study, there are other patient characteristics that may influence patients’ experiences of HF care. Future research on barriers and facilitators to HF care in India could consider using purposive sampling to recruit HF patients with different durations and severity of illness and diversity by socio-economic status and residence, which could generate new insights on the differences or similarities of experiences according to these characteristics. There was a potential limitation of simultaneous translation of audio recordings, however, we did quality checks and retained a few phrases in the regional language (Malayalam) that would lose meaning if translated. The over representation of male patients in our study may have influenced our ability to identify appropriately the traditional gender roles within societies and its potential impact patient management and caregiver support. However, to mitigate this, we asked questions related to male-female differences in HF care during the in-depth interviews with HCPs. As the study was done in a tertiary care specialty public hospital, the results might not be transferable to other types of hospitals like private health care facilities. However, we have included the perspectives of health care providers from private hospitals who were involved in quality improvement initiatives in HF management in their hospital settings. Finally, incorporating the perspectives of patients and caregivers who seek treatment from private care facilities could have enriched our study findings.

## Data availability

### Underlying data

Figshare: Interview transcripts of TIME-HF project. https://doi.org/10.6084/m9.figshare.12763616 ^28^

The file ‘Interview transcripts of TIME-HF project” contains the following underlying data:

- FGD transcripts of patients and caregivers (deidentified)
- Interview transcripts of patients (deidentified)
- Interview transcripts of caregivers (deidentified)
- Interview transcripts of doctors (deidentified)
- Interview transcripts of nurses (deidentified)

### Extended data

Figshare: Interview and FGD guide. https://doi.org/10.6084/m9.figshare.12760307 ^25^

The file “Interview and FGD guide” contains the following extended data:

- Interview & FGD guide.docx (The interview and FGD guide used for patients, caregivers and health care providers.

### Reporting guidelines

Figshare: COREQ checklist for “Patient, caregiver and health care provider perspectives on barriers and facilitators to heart failure care in Kerala: A qualitative study". https://doi.org/10.6084/m9.figshare.12865226.v1 ^27^

Data are available under the terms of the Creative Commons Attribution 4.0 International license (CC-BY 4.0).

## References

[1] Ziaeian B Fonarow GC: Epidemiology and aetiology of heart failure. *Nat Rev Cardiol.* 2016;13(6):368–378. 10.1016/s0195-668x(03)00112-x 26935038 PMC4868779

[2] Gaziano TA Bitton A Anand S: Growing epidemic of coronary heart disease in low- and middle-income countries. *Curr Probl Cardiol.* 2010[cited 2020 Jun 10];35(2):72–115. 10.1016/j.cpcardiol.2009.10.002 20109979 PMC2864143

[3] Huffman MD Prabhakaran D: Heart failure: epidemiology and prevention in India. *Natl Med J India.* 2010;23(5):283–8. 21250584 PMC3913650

[4] India State-Level Disease Burden Initiative CVD Collaborators: The changing patterns of cardiovascular diseases and their risk factors in the states of India: the Global Burden of Disease Study 1990-2016. *Lancet Glob Health.* 2018[cited 2020 Apr 3];6(12):e1339–51. 10.1016/S2214-109X(18)30407-8 30219317 PMC6227386

[5] Sanjay G Jeemon P Agarwal A: In-Hospital and Three-Year Outcomes of Heart Failure Patients in South India: The Trivandrum Heart Failure Registry. *J Card Fail.* 2018[cited 2020 Apr 3];24(12):842–8. 10.1016/j.cardfail.2018.05.007 29885494 PMC7263011

[6] Fathima FN Kahn JG Krishnamachari S: Productivity losses among individuals with common mental illness and comorbid cardiovascular disease in rural Karnataka, India. *Int J Noncommun Dis.* 2019[cited 2020 Aug 24];4(3):86–92. 10.4103/jncd.jncd_17_19 32411923 PMC7221215

[7] Bywaters E Calvert S Eccles S: Safe handover : safe patients. London: British Medical Association;2004[cited 2020 Sep 7]. Reference Source

[8] Gwaltney C Pro S Slagle AF: Hearing the voice of the heart failure patient: key experiences identified in qualitative interviews. *Br J of cardiol.* 2012;19:25. 10.5837/bjc.2012.004

[9] von Schwarz ER He M Bharadwaj P: Palliative Care Issues for Patients With Heart Failure. *JAMA Netw Open.* 2020[cited 2020 Jun 18];3(2):e200011. 10.1001/jamanetworkopen.2020.0011 32101301

[10] Ruppar TM Cooper PS Mehr DR: Medication Adherence Interventions Improve Heart Failure Mortality and Readmission Rates: Systematic Review and Meta-Analysis of Controlled Trials. *J Am Heart Assoc.* 2016; [cited 2020 Jun 18];5(6):e002606. 10.1161/JAHA.115.002606 27317347 PMC4937243

[11] Oertle M Bal R: Understanding non-adherence in chronic heart failure: a mixed-method case study. *Qual Saf Health Care.* 2010[cited 2020 Jun 18];19(6):e37. 10.1136/qshc.2009.033563 21127097

[12] Albert NM Barnason S Deswal A: Transitions of care in heart failure: a scientific statement from the American Heart Association. *Circ Heart Fail.* 2015[cited 2020 Jun 18];8(2):384–409. 10.1161/HHF.0000000000000006 25604605

[13] Carney RM Freedland KE Sheline YI: Depression and coronary heart disease: a review for cardiologists. *Clin Cardiol.* 1997[cited 2020 Jun 18];20(3):196–200. 10.1002/clc.4960200304 9068903 PMC6655605

[14] Nordfonn OK Morken IM Bru LE: Patients' experience with heart failure treatment and self-care-A qualitative study exploring the burden of treatment. *J Clin Nurs.* 2019;28(9–10):1782–93. 10.1111/jocn.14799 30667120

[15] Kennedy BM Jaligam V Conish BK: Exploring Patient, Caregiver, and Healthcare Provider Perceptions of Caring for Patients With Heart Failure: What Are the Implications? *Ochsner J.* 2017;17(1):93–102. 28331455 PMC5349645

[16] Browne S Macdonald S May CR: Patient, carer and professional perspectives on barriers and facilitators to quality care in advanced heart failure. *PLoS One.* 2014;9(3):e93288. 10.1371/journal.pone.0093288 24676421 PMC3968134

[17] Wingham J Frost J Britten N: Needs of caregivers in heart failure management: A qualitative study. *Chronic Illn.* 2015[cited 2020 Jun 18];11(4):304–19. 10.1177/1742395315574765 25795144 PMC4638312

[18] Dunbar SB Clark PC Quinn C: Family influences on heart failure self-care and outcomes. *J Cardiovasc Nurs.* 2008[cited 2020 Jun 18];23(3):258–65. 10.1097/01.JCN.0000305093.20012.b8 18437068 PMC2744587

[19] Agarwal A Davies D Goenka S: Facilitators and barriers of heart failure care in Kerala, India: A qualitative analysis of health-care providers and administrators. *Indian Heart J.* 2019;71(3):235–41. 10.1016/j.ihj.2019.04.009 31543196 PMC6796633

[20] Clark AM: Qualitative research: what it is and what it can contribute to cardiology in the young. *Cardiol Young.* 2009;19(2):131–4. 10.1017/S1047951109003746 19272205

[21] Curry LA Nembhard IM Bradley EH: Qualitative and mixed methods provide unique contributions to outcomes research. *Circulation.* 2009[cited 2020 Jun 18];119(10):1442–52. 10.1161/CIRCULATIONAHA.107.742775 19289649

[22] Ritchie JB Lewis J Nicholls CM: Qualitative Research Practice: A Guide for Social Science Students and Researchers.2013. Reference Source

[23] Hennink M Hutter I Bailey A: Qualitative Research Methods. SAGE;2020;377.

[24] Biernacki P Waldorf DS: Snowball Sampling: Problems and Techniques of Chain Referral Sampling. *sociological methods and research.* 1981;10(2):141–163. 10.1177/004912418101000205

[25] Jose P: Interview and FGD guide.2020[cited 2020 Aug 16]. 10.6084/m9.figshare.12760307

[26] Tong A Sainsbury P Craig J: Consolidated criteria for reporting qualitative research (COREQ): a 32-item checklist for interviews and focus groups. *Int J Qual Health Care.* 2007[cited 2020 Apr 7];19(6):349–57. 10.1093/intqhc/mzm042 17872937

[27] Jose P: COREQ_32 item checklist.2020[cited 2020 Aug 26]. 10.6084/m9.figshare.12865226.v1

[28] Jose P: Interview transcripts of TIME-HF project.2020[cited 2020 Aug 16]. 10.6084/m9.figshare.12763616

[29] Fereday J Muir-Cochrane E: Demonstrating Rigor Using Thematic Analysis: A Hybrid Approach of Inductive and Deductive Coding and Theme Development. *Int J Qual Methods.* 2006[cited 2020 Jul 1];5(1):80–92. 10.1177/160940690600500107

[30] Boyatzis RE: Transforming qualitative information: Thematic analysis and code development. Thousand Oaks, CA, US: Sage Publications, Inc; (Transforming qualitative information: Thematic analysis and code development).1998;xvi:184. Reference Source

[31] Crabtree BF Miller WL: A template approach to text analysis : developing and using codebooks.1992[cited 2020 Jul 1];93–103. Reference Source

[32] McLeroy KR Bibeau D Steckler A: An ecological perspective on health promotion programs. *Health Educ Q.* 1988;15(4):351–77. 10.1177/109019818801500401 3068205

[33] Ylimäki EL Kanste O Heikkinen H: The effects of a counselling intervention on lifestyle change in people at risk of cardiovascular disease. *Eur J Cardiovasc Nurs.* 2015[cited 2020 Aug 4];14(2):153–61. 10.1177/1474515114521725 24463729

[34] Kähkönen O Kankkunen P Saaranen T: Motivation is a crucial factor for adherence to a healthy lifestyle among people with coronary heart disease after percutaneous coronary intervention. *J Adv Nurs.* 2015;71(10):2364–73. 10.1111/jan.12708 26084708

[35] Oscalices MIL Okuno MFP Lopes MCBT: Discharge guidance and telephone follow-up in the therapeutic adherence of heart failure: randomized clinical trial. *Rev Lat Am Enfermagem.* 2019[cited 2020 Aug 4];27:e3159. 10.1590/1518-8345.2484.3159 31432915 PMC6703101

[36] Lifestyle Changes for Heart Failure. www.heart.org. [cited 2020 Apr 10]. Reference Source

[37] Hearn J Ssinabulya I Schwartz JI: Self-management of non-communicable diseases in low- and middle-income countries: A scoping review. *PLoS One.* 2019[cited 2020 Sep 8];14(7):e0219141. 10.1371/journal.pone.0219141 31269070 PMC6608949

[38] Seid MA Abdela OA Zeleke EG: Adherence to self-care recommendations and associated factors among adult heart failure patients. From the patients' point of view. *PLoS One.* 2019[cited 2020 Apr 10];14(2):e0211768. 10.1371/journal.pone.0211768 30730931 PMC6366768

[39] Huffman MD Mohanan PP Devarajan R: Effect of a Quality Improvement Intervention on Clinical Outcomes in Patients in India With Acute Myocardial Infarction: The ACS QUIK Randomized Clinical Trial. *JAMA.* 2018;319(6):567–78. 10.1001/jama.2017.21906 29450524 PMC5838631

[40] Deo MG: "Doctor population ratio for India - the reality". *Indian J Med Res.* 2013[cited 2020 Apr 15];137(4):632–5. 23703329 PMC3724242

[41] Harikrishnan S Sanjay G Anees T: Clinical presentation, management, in-hospital and 90-day outcomes of heart failure patients in Trivandrum, Kerala, India: the Trivandrum Heart Failure Registry. *Eur J Heart Fail.* 2015[cited 2020 Apr 3];17(8):794–800. 10.1002/ejhf.283 26011246

[42] Strömberg A Mårtensson J Fridlund B: Nurse-Led Heart Failure Clinics Improve Survival and Self-Care Behaviour in Patients with Heart Failure: Results from a Prospective, Randomised Trial. *Eur Heart J.* 2003 24(1):1014–23. 10.1016/s0195-668x(03)00112-x 12788301

[43] Rutledge T Reis VA Linke SE: Depression in heart failure a meta-analytic review of prevalence, intervention effects, and associations with clinical outcomes. *J Am Coll Cardiol.* 2006[cited 2020 Apr 11];48(8):1527–37. 10.1016/j.jacc.2006.06.055 17045884

[44] Dekker RL: Patient perspectives about depressive symptoms in heart failure: a review of the qualitative literature. *J Cardiovasc Nurs.* 2014[cited 2020 Apr 14];29(1):E9–15. 10.1097/JCN.0b013e318273a5d6 23151836 PMC3586756

[45] Liguori I Russo G Curcio F: Depression and chronic heart failure in the elderly: an intriguing relationship. *J Geriatr Cardiol JGC.* 2018[cited 2020 Apr 11];15(6):451–9. 10.11909/j.issn.1671-5411.2018.06.014 30108618 PMC6087518

[46] Moraska AR Chamberlain AM Shah ND: Depression, healthcare utilization, and death in heart failure: a community study. *Circ Heart Fail.* 2013;6(3):387–94. 10.1161/CIRCHEARTFAILURE.112.000118 23512984 PMC3689209

[47] Chung ML Pressler SJ Dunbar SB: Predictors of depressive symptoms in caregivers of patients with heart failure. *J Cardiovasc Nurs.* 2010[cited 2020 Apr 11];25(5):411–9. 10.1097/JCN.0b013e3181d2a58d 20714239 PMC2924771

[48] Dionne-Odom JN Hooker SA Bekelman D: Family caregiving for persons with heart failure at the intersection of heart failure and palliative care: a state-of-the-science review. *Heart Fail Rev.* 2017[cited 2020 Apr 14];22(5):543–57. 10.1007/s10741-017-9597-4 28160116 PMC5544594

[49] Wagner EH Austin BT Davis C: Improving chronic illness care: translating evidence into action. *Health Aff (Millwood).* 2001[cited 2020 Jan 30];20(6):64–78. 10.1377/hlthaff.20.6.64 11816692

