# Supplementary material for: Patient, caregiver, and health care provider perspectives on barriers and facilitators to heart failure care in Kerala, India: A qualitative study
Source: Wellcome Open Res. 2021 Apr 19;5:250. Originally published 2020 Oct 22. [Version 2] doi: 10.12688/wellcomeopenres.16365.2 (PMC8078213; doi:10.12688/wellcomeopenres.16365.2)
Supplement: Peer review history

# Peer review: Reviewer response for version 2

**DOI:** 10.21956/wellcomeopenres.18505.r43521

**Published:** 2021-04-26

**Authors:** Deepak Kamath

**Affiliations:** 1 Department of Pharmacology, St. John’s National Academy of Health Sciences, Bangalore, India; 2 DBT/Wellcome Trust India Alliance, Banjara Hills, Hyderabad, India

I have gone through the responses and find them to be satisfactory. Further, I also approve of the changes made to the manuscript.

Is the work clearly and accurately presented and does it cite the current literature?

Yes

If applicable, is the statistical analysis and its interpretation appropriate?

Not applicable

Are all the source data underlying the results available to ensure full reproducibility?

Yes

Is the study design appropriate and is the work technically sound?

Yes

Are the conclusions drawn adequately supported by the results?

Yes

Are sufficient details of methods and analysis provided to allow replication by others?

Partly

Reviewer Expertise:

cardiovascular disease prevention, randomized controlled trials, mixed-methods research, selfcare and medication adherence

I confirm that I have read this submission and believe that I have an appropriate level of expertise to confirm that it is of an acceptable scientific standard.

# Peer review: Reviewer response for version 1

**DOI:** 10.21956/wellcomeopenres.17999.r42779

**Published:** 2021-03-16

**Authors:** Deepak Kamath

**Affiliations:** 1 Department of Pharmacology, St. John’s National Academy of Health Sciences, Bangalore, India; 2 DBT/Wellcome Trust India Alliance, Banjara Hills, Hyderabad, India

I read your article pertaining to the qualitative exploration of the barriers and facilitators of heart failure care with great interest. I do believe that this article represents an original contribution to the factors determining quality of care among heart failure patients. Further, the results will help prioritize some domains within our healthcare system, which if addressed, can ensure high quality heart failure care in Indian settings. The methods used are robust and the reporting is in accordance with the COREQ-32 guideline. 

 I have the following suggestions to make to help improve the article: 

**Background:**

- First para line 6 – Please correct typo – “Within in…” 

**Methods:**

- Sampling HF patients by duration of illness (having a longer lived experience of heart failure) and by socio-economic status and area of residence (urban versus rural) should have been carried out. This should be included as a limitation. 
- Since the patient can be accompanied by more than one relative, how were ‘caregivers’ identified and chosen? 
- Researcher reflexivity – what were the underlying assumptions or underpinning theories that the researchers started with [or conversely, were there no underlying assumptions ('tabula rasa')?] and are there any biases to declare? They may be briefly mentioned either in Methods or in the Results. 
- MD Physicians may manage acute heart failure in semi-urban or rural areas. GPs/family doctors may also be important sources of information. Since they weren’t a part of the sample, this may be mentioned in the discussion. 

**Results:**

- 14 interviews were conducted, but number of patients are 22? This is unclear; were these interviews conducted as dyads? If yes, how many dyads were interviewed? How many caregivers were interviewed? Or were the rest included as a part of FGDs? Please clarify numbers in the first para of the Results section. 
- 19 of 22 were male patients; males are over-represented despite purposive sampling – the implications of this need to be discussed. For instance, traditional gender roles within families/societies will impact patient management and caregiver support.
- Study themes, line 11 – “importance of HCP….” may be changed to “involvement of HCP…”. 
- Coding tree will need to included as a table/figure in accordance with the COREQ32 guidelines. 

**Discussion:**

- The first two paragraphs are essentially a repetition of the Results. This could be put more succinctly and combined into one para. 
- Para 3 last sentence -  The problem of not reaching target GDMT doses is an important one and a bit more elaboration on the role of task sharing/shifting in achieving this goal should be discussed in light of the findings of this study and taking the example of HF clinics in developed countries. 
- I think the over-representation of male patients in the sample and conversely, the under-representation of males as caregivers is a key limitation. Gender roles play an important role in all societies and especially in South Asian countries. This finding need to be contextualized in this regard.

Is the work clearly and accurately presented and does it cite the current literature?

Yes

If applicable, is the statistical analysis and its interpretation appropriate?

Not applicable

Are all the source data underlying the results available to ensure full reproducibility?

Yes

Is the study design appropriate and is the work technically sound?

Yes

Are the conclusions drawn adequately supported by the results?

Yes

Are sufficient details of methods and analysis provided to allow replication by others?

Partly

Reviewer Expertise:

cardiovascular disease prevention, randomized controlled trials, mixed-methods research, selfcare and medication adherence.

I confirm that I have read this submission and believe that I have an appropriate level of expertise to confirm that it is of an acceptable scientific standard, however I have significant reservations, as outlined above.

# Response

**Published:** 2021-03-30

**Authors:** Panniyammakal Jeemon

**Affiliations:** Sree Chitra Tirunal Institute for Medical Sciences and Technology, Trivandrum, India

Thank you very much for sharing your thoughtful comments. We greatly appreciate the comments on our manuscript, and we feel that they have helped us to further strengthen the manuscript. Please find below our detailed response to your comments. 

**Background: First para line 6 – Please correct typo – “Within in…”**

              

 We have changed to “Within India”.

**2.Methods:**

**2.1 Sampling HF patients by duration of illness (having a longer lived experience of heart failure) and by socio-economic status and area of residence (urban versus rural) should have been carried out. This should be included as a limitation.**

 Thank you for the comment. We have added a couple of sentences  in the “Strengths and limitations” section: “Although we used purposive sampling to ensure inclusion of HF patients of varying ages in our study, there are other patient characteristics that may influence patients’ experiences of HF care. Future research on barriers and facilitators to HF care in India could consider using purposive sampling to recruit HF patients with different durations and severity of illness and diversity by socio-economic status and residence, which could generate new insights on the differences or similarities of experiences according to these characteristics."

**2.2 Since the patient can be accompanied by more than one relative, how were ‘caregivers’ identified and chosen?**

 We have added a sentence in the “Study participants, recruitment, and sampling” section as follows: “When a patient was accompanied by more than one caregivers, the primary caregiver was chosen as the person who is most involved in care, as identified by the patient.”

 ​​​​​​ **Researcher reflexivity – what were the underlying assumptions or underpinning theories that the researchers started with [or conversely, were there no underlying assumptions ('tabula rasa')?] and are there any biases to declare? They may be briefly mentioned either in Methods or in the Results.**

 We appreciate this query. We used an inductive approach to analysis and did not start with underpinning theories. We have revised the sentence in the “data analysis” part of methods section that “The researchers practiced reflexivity throughout the study and remained sensitive to the ways in which their experiences and prior assumptions shaped the findings.”

**MD Physicians may manage acute heart failure in semi-urban or rural areas. GPs/family doctors may also be important sources of information. Since they weren’t a part of the sample, this may be mentioned in the discussion.**

 In this study, we recruited health care providers who were cardiologists or senior residents in the Department of Cardiology with an MD qualification who worked particularly in HF failure management. This was decided to identify information-rich health care providers who had specific experience in managing patients and caregivers with heart failure. We did not intend to involve GPs/family doctors in the study as it was done to improve the quality of care in tertiary care settings. 

**Results:**

**3.1 14 interviews were conducted, but number of patients are 22? This is unclear; were these interviews conducted as dyads? If yes, how many dyads were interviewed? How many caregivers were interviewed? Or were the rest included as a part of FGDs? Please clarify numbers in the first para of the Results section.**

 We would like to clarify that we did not focus on the patient-caregiver dyad in the current study. We had 10 in-depth interviews and two focus group discussions (with six participants each) with patients. Hence, the total number of patients included in in-depth interviews and focus group discussions in the study were 22. We had 4 in-depth interviews and one focus group discussions (with 5 participants) with the caregivers. Hence, the total number of caregivers in the in-depth interviews and focus group discussions in the study was 9.  We have updated the results section accordingly. 

 ​​​​​​​

**19 of 22 were male patients; males are over-represented despite purposive sampling – the implications of this need to be discussed. For instance, traditional gender roles within families/societies will impact patient management and caregiver support.**

 Thank you for the comment. We agree that we had an overrepresentation of male patients in the study. Also, this overrepresentation could have limited our ability to identify appropriately the traditional gender roles within societies and its impact on patient management and caregiver support. We accept it as a limitation and have also added this in the strengths and limitation section as follows:

 "The over representation of male patients in our study  may have influenced our ability to identify appropriately the traditional gender roles within societies and its potential impact patient management and caregiver support. However, to mitigate this, we asked questions related to male-female differences in HF care during the in-depth interviews with HCPs. .”

 ​​​​​​​ **Study themes, line 11 – “importance of HCP….” may be changed to “involvement of HCP…”. **We have changed to “involvement of HCP”

 ​​​​​​​ **Coding tree will need to included as a table/figure in accordance with the COREQ 32 guidelines. **We appreciate the comment. The data were analysed using thematic analysis by a hybrid approach which incorporating the data-driven approach from Boyatzis 1998, and the deductive a priori template of codes approach by Crabtree and Miller 1999, which do not mandate a coding tree for the data analysis process. Thus, we have not included a coding tree.

**4.Discussion:**

 ​​​​​​​ **The first two paragraphs are essentially a repetition of the Results. This could be put more** **succinctly and combined into one para.**

 We have shortened the initial paragraphs of the discussion section. Further, it is a standard practice to summarise the key findings in the first paragraph of the discussion section. 

 ​​​​​​​

**Para 3 last sentence -  The problem of not reaching target GDMT doses is an important one** **and a bit more elaboration on the role of task sharing/shifting in achieving this goal should**

**be discussed in light of the findings of this study and taking the example of HF clinics in**

**developed countries.**

 We have modified the section in discussion part as follows:

 “The Trivandrum Heart Failure Registry ^40^ of 1205 HF cases in Kerala reported that GDMT was given only to 19% and 25% of patients with left ventricular systolic dysfunction during hospitalization and at the time of discharge, respectively. Quality improvement initiatives like task sharing with non-physician health workers may improve prescription and adherence to GDMT and help the patients to reach the target dose for each drugs based on evidence and clinical recommendations. For example, Strömberg et al ^49^ reported that task sharing strategies such as nurse-led heart failure clinics improved the survival and reduced the number of readmissions for patients with HF.”

 ​​​​​​​ **I think the over-representation of male patients in the sample and conversely, the underrepresentation of males as caregivers is a key limitation. Gender roles play an important role in all societies and especially in South Asian countries. This finding need to be contextualized in this regard.**

 Thank you for this important comment. We have explained about this in response a comment above. We have also added this as a limitation of study.

# Peer review: Reviewer response for version 1

**DOI:** 10.21956/wellcomeopenres.17999.r41208

**Published:** 2020-12-02

**Authors:** Mohammed Assen Seid

**Affiliations:** 1 Department of Clinical Pharmacy, University of Gondar, Gondar, Ethiopia

Thank you giving me the chance to review this interesting paper. The paper focus on an important issue which will help to explore challenges for heart failure management. Overall the paper may need some minor revisions to be indexed.  

 I have these comments and suggestions:  

1. In the abstract, correct aims to ‘aimed’.
2. In the background it says: "globally, an estimated 26 million…". This needs updating as it is now about 37 million ^1^.
3. In the method section it is not clear that how you selected the caregivers. In order to discuss the influence of the caregiver for self-management of HF you need to participate each patients with their respective caregivers.
4. You may need to justify the sampling technique you used (Purposive sampling, snowball, etc.).
5. You include limited number of health care providers (Cardiologists, Nurses, etc. - what about other health care providers, like pharmacists, who discuss more about their self-management with HF patients?).
6. Your discussion seems to report known facts rather than elaborating your main findings; it would be better to focus on your findings.
7. It says " *Support from caregivers was key for promoting patients’ adherence to optimal HF care’" *I don’t think so...you have measured their adherence in respective of their caregiver influence? Otherwise you may need to cite the reference of this evidence.

Is the work clearly and accurately presented and does it cite the current literature?

Yes

If applicable, is the statistical analysis and its interpretation appropriate?

Yes

Are all the source data underlying the results available to ensure full reproducibility?

Yes

Is the study design appropriate and is the work technically sound?

Yes

Are the conclusions drawn adequately supported by the results?

Yes

Are sufficient details of methods and analysis provided to allow replication by others?

Yes

Reviewer Expertise:

Non communicable chronic disease (Heart failure, HTN, Diabetes...etc) and infectious disease

I confirm that I have read this submission and believe that I have an appropriate level of expertise to confirm that it is of an acceptable scientific standard.

## References

[1] : Epidemiology and aetiology of heart failure. *Nature Reviews Cardiology*.2016;13(6) : 10.1038/nrcardio.2016.25 368-378 10.1038/nrcardio.2016.25 PMC486877926935038

# Response

**Published:** 2021-03-30

**Authors:** Panniyammakal Jeemon

**Affiliations:** Sree Chitra Tirunal Institute for Medical Sciences and Technology, Trivandrum, India

Thank you very much for sharing your comments. We greatly appreciate the thoughtful comments on our manuscript, and they have helped us to further strengthen the manuscript. Please find below our point-by-point response to your comments. 

**In the abstract, correct aims to ‘aimed’.**

 We have changed to “aimed”

**In the background it says: "globally, an estimated 26 million…". This needs updating as it is** **now about 37 million** **1** **.**

              

 The sentence is revised to "Globally,  more than 37.7 million people suffer from heart failure ^1 ^."

**In the method section it is not clear that how you selected the caregivers. In order to** **discuss the influence of the caregiver for self-management of HF you need to participate** **each patients with their respective caregivers.**

 Thank you for this comment. We recruited caregivers who accompanied each patient for their hospital consultation using purposive sampling. We have stated in the study participant, recruitment and sampling section that “Caregivers who accompanied patients for hospital consultations were also invited to participate in the study.”

 The goal of our study was also to understand the barriers and facilitators of heart failure care from caregiver’s perspective. However, we did not intend to focus on the patient-caregiver dyad in the current study.  We agree that exploring the barriers and facilitators for heart failure care among patient-caregiver dyads can be a potential future line of research. 

**You may need to justify the sampling technique you used (Purposive sampling, snowball,** **etc.).**

**For patients:** Purposive sampling was utilized to enable a diversity of characteristics across age and gender. We have stated in “Study participants, recruitment, and sampling” section that “Purposive sampling ^22^ was used to recruit patients and caregivers across age and gender groups to obtain a diverse characteristic and this has been widely used in qualitative research.”

**For health care providers:**  We utilized both purposive sampling and snowball sampling. We revised the “Study participants, recruitment, and sampling” section as follows:

 Firstly, purposive sampling [22] was utilized to identify information-rich individuals with diverse characteristics (e.g., type of health care provider) in our study. We also included HCPs who were experienced cardiologists working in private hospitals of central Kerala.  Secondly, to identify HCPs with specific experience in HF management, snowball recruitment [24] was used by asking HCPs at the end of interviews to refer other HCPs. They were subsequently contacted and invited to participate in the study. 

**You include limited number of health care providers (Cardiologists, Nurses, etc. – what about other health care providers, like pharmacists, who discuss more about their self-management with HF patients?).**

 This study was performed in the heart failure clinic of a tertiary care hospital in Kerala, India, where cardiologists and nurses are the predominant team members. We agree that involving clinical pharmacists would have enriched the findings. However, there is a limited number of clinical pharmacists in the current team, which is a potential limitation in heart failure management in Kerala.

**Your discussion seems to report known facts rather than elaborating your main findings; it** **would be better to focus on your findings.**

 We have revised the discussion section based on your comments.

**It says " *Support from caregivers was key for promoting patients’ adherence to optimal HF care’" *I** **don’t think so...you have measured their adherence in respective of their caregiver influence? Otherwise, you may need to cite the reference of this evidence.**

 Thank you for this comment. It is a finding from our study. As a standard practice, we have summarised the key findings in the first paragraph of the discussion section. For brevity, we have also revised the sentence as follows:

 “Heart failure patients in general appreciated the role of caregivers and it was perceived as important for their adherence to heart failure treatment in our study.”
